# Tackling Inequalities in Oral Health: Bone Augmentation in Dental Surgery through the 3D Printing of Poly(ε-caprolactone) Combined with 20% Tricalcium Phosphate

**DOI:** 10.3390/biology12040536

**Published:** 2023-03-31

**Authors:** Nicola De Angelis, Andrea Amaroli, Maria Giovanna Sabbieti, Alessia Cappelli, Alberto Lagazzo, Claudio Pasquale, Fabrizio Barberis, Dimitrios Agas

**Affiliations:** 1 Department of Surgical and Diagnostic Sciences (DISC), University of Genoa, 16132 Genoa, Italy; 2 Department of Civil, Chemical and Environmental Engineering (DICCA), University of Genoa, 16100 Genoa, Italy; 3 Faculty of Dentistry Department of Periodontology, Trisakti University, Jakarta 11440, Indonesia; 4 Department of Earth, Environmental and Life Sciences (DISTAV) University of Genoa, 16132 Genoa, Italy; 5 School of Biosciences and Veterinary Medicine, University of Camerino, 62032 Camerino (MC), Italy

**Keywords:** additive manufacturing, rapid prototyping, polymer, dentition, oral disorder, bone defect, socket, bone augmentation

## Abstract

**Simple Summary:**

Personalized medicine and overcoming healthcare inequalities have gained significant popularity in recent years. Polymers offer an ideal solution due to their cost-effectiveness, ease of customized 3D printing, and potential for wide-scale expansion. Polymers blended with β-tricalcium phosphate (TCP) have been found to synergize with the environmental tissues of maxillary bones and promote osteoconductivity. However, little is known about their properties after printing and their ability to maintain their biological role; additionally, limitations exist in 3D printing when high TPC concentrations are added. Our study demonstrated that poly ε-caprolactone (PCL)+β-TCP 20% composite can be successfully printed and is a suitable material for commercial 3D printing. The material also demonstrated biocompatibility, supporting osteoblast adhesion and promoting cell proliferation and differentiation. The composite can also sustain ISO14937:200935 sterilization procedures, which makes it an ideal material for printing medical devices that can be used by clinicians worldwide.

**Abstract:**

The concept of personalized medicine and overcoming healthcare inequalities have become extremely popular in recent decades. Polymers can support cost reductions, the simplicity of customized printing processes, and possible future wide-scale expansion. Polymers with β-tricalcium phosphate (TCP) are well known for their synergy with oral tissues and their ability to induce osteoconductivity. However, poor information exists concerning their properties after the printing process and whether they can maintain an unaffected biological role. Poly(ε-caprolactone) (PCL) polymer and PCL compounded with TCP 20% composite were printed with a Prusa Mini-LCD-^®^3D printer. Samples were sterilised by immersion in a 2% peracetic acid solution. Sample analyses were performed using infrared-spectroscopy and statical mechanical tests. Biocompatibility tests, such as cell adhesion on the substrate, evaluations of the metabolic activity of viable cells on substrates, and F-actin labelling, followed by FilaQuant-Software were performed using a MC3T3-E1 pre-osteoblasts line. PCL+β-TCP-20% composite is satisfactory for commercial 3D printing and appears suitable to sustain an ISO14937:200937 sterilization procedure. In addition, the proper actin cytoskeleton rearrangement clearly shows their biocompatibility as well as their ability to favour osteoblast adhesion, which is a pivotal condition for cell proliferation and differentiation.

## 1. Introduction

Healthy dentition is valuable for the maintenance of arch length, aesthetics, and phonetics, the prevention of oral environment imbalance, altered mastication, and, therefore, the insurgence of associated diseases (such as systemic infections, digestive disorders, cancer, improper nutritional condition, and age-related neurodegeneration) [1]. Indeed, oral health support may promote survival in older adults, both through sustaining healthy and functional teeth and through targeted interventions for the maintenance/replacement of complete dentition [2].

It has been projected that 8.6 million individuals in the United States of America will suffer from edentulism in the year 2050 [3], and the effect will be much greater in developing countries [4]. In accordance with WHO guidelines [5], persons in the age group from 35 to 45 years exhibit the maximum prevalence of partial edentulousness, and the condition can rapidly evolve to total edentulousness at older ages due to a lack of dental treatment.

The phenomenon is a consequence of oral healthcare inequalities in third-world countries, but it is also dramatically experienced in Europe and North America, depending on people’s socioeconomic status [4,6,7].

Teeth loss that is a consequence of traumas or associated with oral disorders, such as periodontal disease, degeneration following post-traumatic extractions, and cancers of the mouth, may lead to moderate or severe bone deficiencies and represents a global public health challenge [8]. A bone defect is an anatomical condition that does not allow the conventional placement of implants [9]. Therefore, alveolar bone augmentation is frequently required to restore lost anatomy and function.

Presently, bone substitutes and scaffolds are the key materials used in bone augmentation techniques. The most common bone substitutes used in clinical practice are heterologous grafts [10] which have osteoconductive properties, guaranteed constant availability, and a reasonable price, but numerous patients refuse them because of religious faith and ethical lifestyle choices [11].

Moreover, synthetic bone substitutes are employed in dentistry, despite light and shadow being described [12,13,14].

In fact, these synthetic substitutes present excellent biocompatibility and structural integrity for the whole period of their implantation, but their need to be removed through a second surgical procedure and their exposure to the oral environment represent drawbacks that must be carefully considered [15,16].

Additionally, meshes such as titanium barriers must be shaped and adapted to the bone defect profile during surgery [17,18,19]. This sensitive, time-consuming, and laborious step increases the duration of the surgery, and the outcome of the procedure is deeply influenced by the operator’s ability.

The golden standard for bone regeneration continues to be autogenous bone, despite its invasiveness and high morbidity. Therefore, bone grafts and bone substitutes in oral regenerative medicine dramatically impact oral problems that are linked to an individual’s physical, psychological, and social wellbeing in terms of their acceptance before and discomfort after surgical approaches [20,21,22].

Recent efforts have included the identification of natural or synthetic materials approved by the FDA which exhibit improved wound healing and bone formation bioresorbable properties during the healing phase and reduce the need for a second surgical procedure [23,24].

The possibility of employing additive manufacturing processes to fabricate customised devices for bone regeneration has been investigated over the last decade to improve oral disease management [25,26,27].

Indeed, meshes obtained through 3D printing have several surgical advantages, including higher precision without the need for intra-surgical adaptation, plasticity and space-making, and round margins and corners. However, the high costs associated with this printing technique and the materials it employs may represent important limitations [28,29].

Among the different 3D printing techniques, fused deposition modelling (FDM) has the potential to produce, highly reproducible, bioresorbable 3D scaffolds with a fully interconnected pore network at a reasonably low cost [30]. Poly(lactic acid) (PLA) and poly(ε-caprolactone) (PCL) are typical materials employed in FDM which show biodegradable and bioresorbable properties [31]. However, PLA can be considered too brittle for applications requiring high plastic deformations at higher stress levels [32,33] such as those required in dentistry applications.

However, PCL, which is another leading FDA-approved aliphatic polymer, exhibits a low tensile strength (about 23 MPa) but an elevated elongation at break (4700%) which lead to highly elastic behaviour and a smaller Young’s modulus (from 0.2 to 0.4 GPa) than that of PLA [33]. Additionally, PCL has a high biocompatibility which makes it a widely used material in the biomedical field due to its higher stability [33,34,35]. A limitation associated with the use of PCL is related to the time required for its complete degradation. Complete degradation can occur within 2–3 years, which is a relatively long time for many dentistry purposes. Notably, the combination of PCL with hydroxyapatite (HA) or tricalcium phosphate (TCP) drastically decreases its reabsorption time [36].

TCP is well known for its synergy with oral tissues and its ability to induce osteoconductivity. In fact, due to its high mechanical properties and exceptional bone remodelling capacity, TCP can be used along with polymers to create suitable tissue scaffolds [31]. In this work, we focused on the identification of a reliable biocompatible PCL-based material which could be printed through the FDM technique and would show suitable reabsorption properties to avoid a second surgical procedure during odontoiatric bone augmentation.

Therefore, the main scope of our work was to remedy two issues: (i) the necessity to introduce a novel innovative material and consequently rejuvenate surgical practices and (ii) guarantee the chance for dental operators to create the perfectly-fitting shape they need without the necessity of a “removal step”.

An additional objective of our research was to present PCL+TCP polymer material which is known to be highly performing in its mechanical and biological dimensions as an efficient biomaterial fused in the 3D printing process.

Particularly, to comply with personalized medicine, overcome healthcare inequalities, and respect the ecological global healthcare system, we identified a non-plastic PCL+beta-TCP 20% (β-TCP 20%) composite-ceramic polymer that could be printed with a commercial, market-available 3D printer. The biomaterial should sustain an ISO14937:2009 sterilisation procedure [37] and allow a suitable adhesion, growth, and matrix organisation of MC3T3-E1 preosteoblasts cells.

## 2. Materials and Methods

### 2.1. Sample Materials, Sample Geometries, and FDM Process

To comply with our research purposes, PCL polymer and PCL compounded with β-TCP 20% composite polymer were produced by the specialised start-up company Nadir s.r.l. (Treviso, Italy). After several preliminary experimental tests performed by the DICCA Lab with Nadir, the percentage of osteoconductive material used was the more balanced compromise with the use of a commercial 3D printer.

Higher percentages could represent a limitation for the extrusion through the printer nozzle. The sample geometry was designed using Thinkercad^®^ software, according to the ISO 178 ASTM D790 standard [38]. Essentially, the software generated a .stl file which could be printed with commercial printers available on the market. The sample dimensions were 70 mm length, 8 mm height, and 3 mm thickness. The generated “.stl” format file was printed with a Prusa Mini LCD ^®^3D printer (Prusa Research a.s., Prague, Czech Republic).

Each printer brand has its own slicer program; therefore, a further elaboration of the CAD-designed file allowed us to decide the features of the sample, such as the infill, the geometry of the infill, and the layer thickness. For more articulated forms, the program enabled the creation of build plates and/or supports for concave parts.

**Figure 1:**
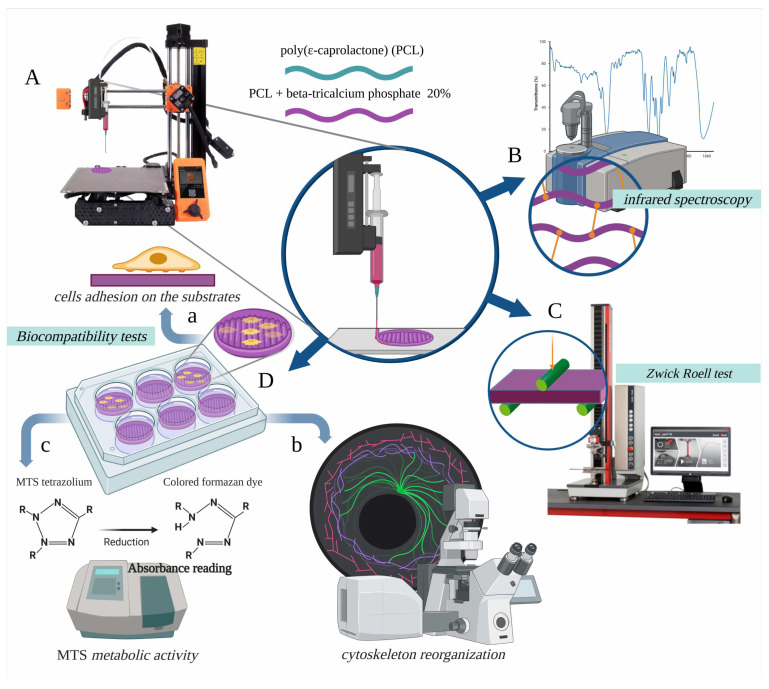
The poly(ε-caprolactone) (PCL) polymer and the PCL compounded with beta-tricalcium phosphate 20% (β-TCP 20%) composite polymer was printed according to the experimental setup shown in (**A**) and sterilised with a 2% peracetic acid solution. The PCL and β-TCP 20% printed samples were photochemically (**B**) and mechanically (**C**) characterised. The biocompatibility of the β-TCP 20% samples (**D**) was tested to show the effects of their interaction with preosteoblast cells. For this, the ability of the cells to adhere to the substrate (**a**), their cytoskeletal re-organisation (**b**), and the activity of cell metabolism (**c**) were assayed.

The printing operation was carried out using a single nozzle following the parameters developed with the material producer which are shown in Table 1. The materials were printed and tested according to the experimental setup described in Figure 1.

### 2.2. Sterilisation of the Samples

The Italian law nr. 46/1997 [39] following the European law 93/42 [40] on medical devices confirms the crucial role of the decontamination and sterilisation of a “critical device”. Among the Standard ISO14937:2009 [35] procedures permitted for sterilisation, autoclave steam, ethylene dioxide, and gamma irradiation cannot be applied to our polymers because they affect their chemical structure and mechanical properties.

Therefore, our samples were decontaminated by immersion in a 2% peracetic acid solution through a “cold sterilisation” procedure. The samples were weighed before and after the sterilisation using a Radwag weight scale (RADWAG, Radom, Poland) to evaluate any possible incorporation of the sterilising agent into the samples.

### 2.3. Sample Analysis by Infrared Spectroscopy

Our sample materials (PCL and PCL+β-TCP 20%) were polyesters that could be hydrolysed during the sterilisation treatment; therefore, the stability of the chemical bonds and, particularly, the ester bonds were analysed through infrared (IR) spectroscopy. IR spectroscopy or vibrational spectroscopy measures the interaction of IR radiation with matter through absorption, emission, or reflection and allows the identification of chemical substances or functional groups in solid, liquid, or gaseous forms. It can be used to characterise new materials or identify and verify known and unknown samples. The IR-attenuated total reflection (ATR) spectra were measured using a Thermo Nicolet Fourier transform (FT-) IR instrument equipped with an ATR accessory (diamond window) (Thermo Fisher Scientific Inc., Lafayette, LA, USA). One hundred scans were made at a 4 cm^−1^ resolution using a deuterated triglycine sulphate (DTGS detector) and background air. The spectra were collected for all the samples in the 4000–400 cm^−1^ range (mid-IR region). The test samples were analysed two hours after the sterilisation process.

### 2.4. Static Mechanical Tests

The particular static test that analysed flexural stress was chosen because it best simulates the mechanical stress due to the masticatory function over the implanted device when the mesh is fixed to the bone [41,42].

The mechanical characterisation allowed the evaluation of important parameters, such as the modulus of elasticity in bending, the flexural strength, and the flexural stress–strain response on our PCL and PCL+β-TCP 20% samples. The reference standard for the material was according to ISO 178—ASTM D790 [36]. The tests were performed on the samples both pre- and post-sterilisation at 23 °C using a Zwick Roell Z0.5 instrument (ZwickRoell Group, Ulm-Einsingen, Germany).

**Figure 2:**
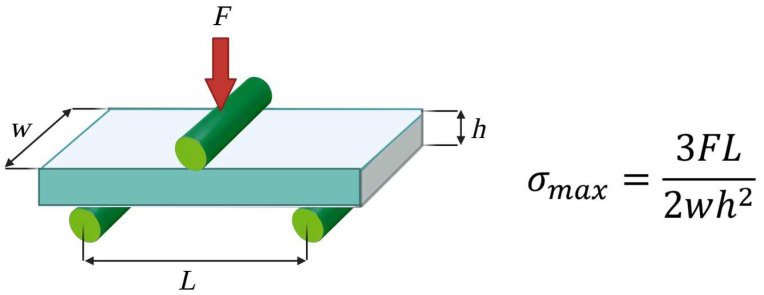
Description of static mechanical tests. The samples were placed on two support anvils and bent through an applied force on one or two anvils to measure their stress–strain data. The beam was proportioned so that it did not fail through shear or by lateral deflection before reaching its ultimate flexural limit. The equation reported for the highest stress at the moment of rupture was employed, where *F* = the load at the bar centre, *L* = the distance between the two lower supports, *w* = the width of the specimen, and *h* = the thickness of the specimen.

A general description of the tests is provided in Figure 2. Once all the necessary data had been registered and sent to the dedicated TestXpert software (ZwickRoell Group, Ulm-Einsingen, Germany), the stress–strain response and parameters, such as the flexural modulus, were calculated.

### 2.5. Biocompatibility Tests: Preosteoblast Cell Line

In vitro characterizations of the 3D printed PCL and PCL+β-TCP 20% samples were performed to assess their biocompatibility and their ability to allow the attachment and growth of cells.

MCT3-E1 subclone 4 cells (mouse calvaria preosteoblasts) were used (ATCC, LGC Standards s.r.l., Milan, Italy) because they show a strong ability to differentiate and mineralise and are considered a good model for studying in vitro osteoblast differentiation. In addition, the behaviour of this cell line is very similar to that of primary calvarial preosteoblasts.

### 2.6. Biocompatibility Tests: Cell Adhesion on the Substrate

The MC3T3-E1 pre-osteoblasts were placed at a density of 5000 cells/cm^2^ into 12 well culture plates (Euroclone, SpA, Milano, Italy) and onto the previously sterilised 3D printed PCL+β-TCP 20% substrates. The cells were grown for four days in a minimum essential medium (α-MEM; Life Technologies, Monza, Italy) which was supplemented with 10% heat-inactivated fetal calf serum (HIFCS) (Life Technologies, Monza, Italy), penicillin, and streptomycin (Sigma Aldrich, Milano, Italy). Control cultures were produced by growing the cells on culture plates as substrates.

Then, all cultures were washed three times with phosphate-buffered saline (PBS) 0.1 M, pH 7.4 and fixed in 4% paraformaldehyde (PFA) which was diluted in PBS for 20 min at room temperature. The cultures were then washed three times with PBS, and the cells were stained with haematoxylin (5 min) and eosin (30 s). Following Dellinger et al. [43], 5% toluidine blue (5 s) was used to point out the extracellular matrix (ECM). After washing with tap water, the substrates were mounted on slides and imaged using a light microscope (Axiophot, Zeiss, Oberkochen, Germany). Image analysis was performed using NIH ImageJ software [44].

### 2.7. Biocompatibility Tests: F-Actin Labelling and FilaQuant Software

The MC3T3-E1 preosteoblasts were treated as described above (cell adhesion on the substrates), and after fixation, they were washed three times in PBS, permeabilised with 0.3% Triton X-100 in PBS for 30 min on ice, and incubated with 0.5% bovine serum albumin (BSA) which was diluted in PBS for 20 min at room temperature. Then, the cultures were incubated with a 4 × 10^−6^ mol/L phalloidin tetramethylrhodamine isothiocyanate (TRITC) conjugate (Sigma-Aldrich, Milan, Italy) which was diluted in PBS for 30 min at room temperature. Finally, the cultures were mounted on slides with 1:1 PBS/glycerol.

The slides were then analysed with a C2 Plus confocal laser scanning microscope (Nikon Instruments, Florence, Italy). The microscope images were converted into a TIFF format and processed using NIS-Elements imaging software (Nikon Instrument, Florence, Italy).

### 2.8. Biocompatibility Tests: Evaluation of the Metabolic Activity of Viable Cells on Substrates (MTS Assay)

The MC3T3-E1 preosteoblasts were plated at a density of 5000 cells/cm^2^ onto the sterilised 3D printed PLC+β-TCP 20% substrates which were contained in 48 well culture plates (Euroclone, SpA, Milano, Italy). The cells were grown for three days to an 80% confluence in α-MEM (Life Technologies, Monza, Italy) and supplemented with 10% HIFCS (Life Technologies, Monza, Italy), penicillin, and streptomycin (Sigma Aldrich, Milan, Italy). The control cultures were produced by growing cells on culture plates as substrates. Then, the cells were incubated with a CellTiter 96 Aqueous One Solution Reagent (Promega Italia Srl, Milan, Italy) for 2 h in a humidified 5% CO_2_ atmosphere. The coloured formazan was measured by reading the absorbance at 490 nm using a Tecan fluorometer (Tecan Italia Srl, Cernusco Sul Naviglio, Milan, Italy).

### 2.9. Statistical Analysis

The statistical analyses were performed using MATLAB software (The Math-Works, Inc. MathWorks 1 Apple Hill Drive, Natick, MA, USA) and compared the means ± standard deviations. Values of *p* < 0.05 were considered significant. The results were representative of those acquired by independent experiments which were repeated at least three times [45].

## 3. Results

### 3.1. Weight Evaluation

All samples were evaluated before and after the sterilisation procedures to identify changes in their weights. As shown in Table 2, the statistical analysis did not show significant differences between the weights (*p* > 0.05).

### 3.2. Infrared Spectroscopy

**Figure 3:**
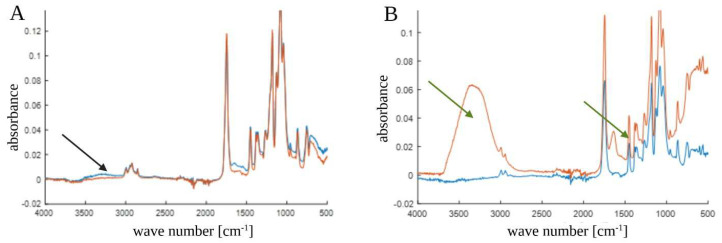
(**A**) Fourier transform infrared (FT-IR) spectrum of poly(ε-caprolactone) (PCL) samples. The black arrow indicates that there was no interaction in the ester group between the unsterilized PCL (blue line) and sterilized PCL (red line) samples. (**B**) FT-IR spectrum of PCL compounded with beta-tricalcium phosphate 20% (PCL+β-TCP 20%) samples. The green arrows indicate the interactions in the ester group between the unsterilized composite (blue line) and the sterilized composite (red line) samples.

All samples that were immersed in the peracetic acid solution were tested. The PCL samples showed an ester group peak at 1750 cm^−1^. No interaction between the solution of peracetic acid and the polymers was highlighted by the analysis (Figure 3A). The absence of any interaction between the ester group and the solution used for the sterilization was also evident.

Different results were revealed in the spectra of the PCL+β-TCP 20% composite polymers (Figure 3B). The polymer bands remained unchanged and evident, but the presence of β-TCP might have induced hydrophilic behavior. Therefore, changes in the spectrum were noticed after sterilization. Particularly, changes occurred in the bands relating to the -OH functional groups (3000–3600, 1500–1670, and below 700 cm^−1^) because of the adsorption of water.

### 3.3. Static Mechanical Tests—Three-Point Bending Zwick Roell Test

The results described above suggest that changes in the mechanical properties of the studied samples occurred. Therefore, destructive three-point bending Zwick Roell tests were performed.

**Figure 4:**
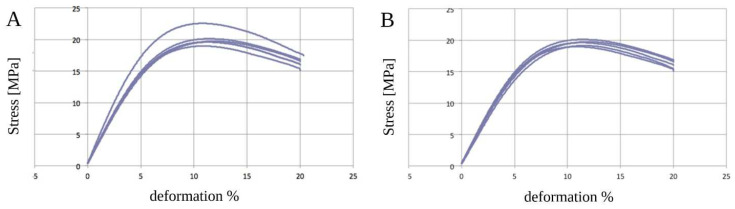
Graphical representations of the five analyses of unsterilised (**A**) and sterilised (**B**) poly(ε-caprolactone) (PCL) samples. No differences in deformation (%) were noticed in the range of observation.

**Figure 5:**
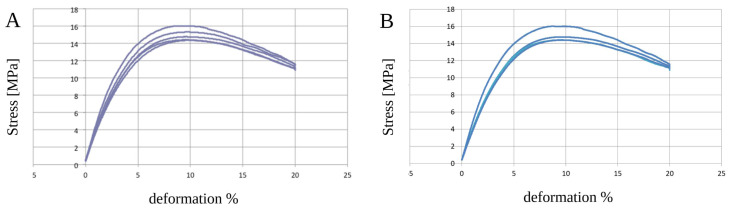
Graphical representations of the five analyses of unsterilized (**A**) and sterilized (**B**) poly(ε-caprolactone) (PCL) compounded with beta-tricalcium phosphate 20% (PCL+β-TCP 20%) samples. No differences in deformation (%) were noticed in the range of observation.

The PCL samples showed an elastic behavior without statistically significant alterations (*p* > 0.05) due to the sterilization process (Table 3 and Figure 4A,B). The composite polymer PCL+β-TCP 20% samples did not show any significant changes (*p* > 0.05) in their mechanical properties (Table 3 and Figure 5A,B).

### 3.4. Biocompatibility Test

**Figure 6:**
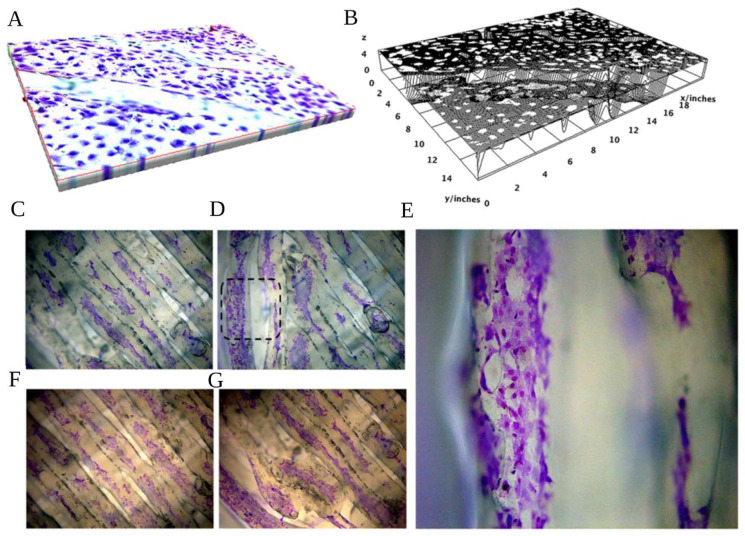
(**A**) Spatial representation of the poly(ε-caprolactone) (PCL) compounded with beta-tricalcium phosphate 20% (PCL+β-TCP 20%)-based biomaterial with toluidine blue-stained preosteoblasts, magnification 20×. (**B**) Surface plot of the PCL+β-TCP 20% compounds with the relevant expansion of white dots (pre-osteoblasts) on almost the whole surface. (**C**) Toluidine blue staining of preosteoblasts expanding on the central meshes or the peripheral (**D**,**E**) meshes of the compound, magnification 20× (**C**,**D**) and 40× (**E**, as magnification of black dashed square of **D**). The hematoxylin/eosin staining of the proliferating preosteoblasts on the biomaterial network better elucidates the affinity of the compound with the developing cells in the centre (**F**) or peripheral parts of the substrate (**G**).

Cell adhesion and viability/proliferation are fundamental parameters for biocompatibility analyses. The cell adhesion onto the PCL+β-TCP 20% substrates was evaluated by hematoxylin/eosin staining. Figure 6 shows that the scaffolds were remarkably suitable for cell attachment. Indeed, the preosteoblasts displayed a high level of adhesion and regular distribution along the scaffold after four days of culture.

**Figure 7:**
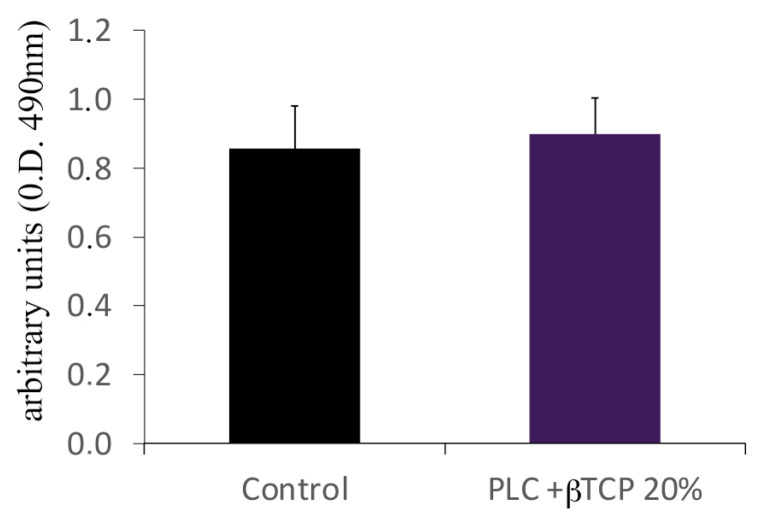
MTS assay. Note the suitability of the poly(ε-caprolactone) (PCL) compounded with beta-tricalcium phosphate 20% (PCL+β-TCP 20%) samples for supporting the growth and viability of the preosteoblasts relative to those cultured in the normal culture dishes. The values from three different experiments were calculated as the means ± the standard deviations. No statistically significant differences were observed (*p* > 0.05).

In addition, the substrates were able to induce cell viability and proliferation on a level comparable to that of the culture plates (Figure 7).

### 3.5. Actin Distribution

**Figure 8:**
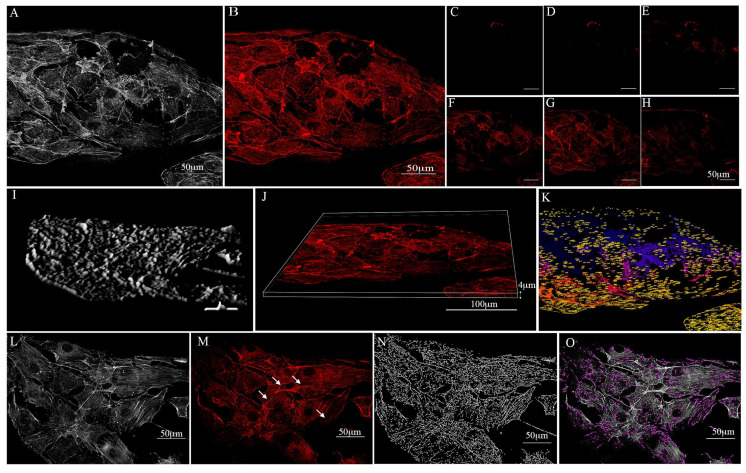
Preosteoblast growth over the poly(ε-caprolactone) (PCL) compounded with beta-tricalcium phosphate 20% (PCL+β-TCP 20%)-based substrate which was stained with phalloidin (TRITC-conjugate) for F-actin detection. (**A**) Map of the minimum of n nearest neighbour cells on the compound. (**B**) Confocal microscopy captured the preosteoblast actin filaments spreading on the surface of the substrate. (**C**–**H**) Confocal microscopy 3D illustration of the phalloidin-labelled F-actin of preosteoblasts over the substrate. Note the F-actin distribution over the substrate in multiple scans. (**I**,**J**) 3D surface plot of the substrate. (**K**) Projections of cell maps on the biomaterial. The numbers in yellow represent the pixel distances between the cultured cells and the substrate. (**L**) A map of proximal neighbour preosteoblasts on the substrate meshes. Note the actin adhesion points (white arrows) between the cells (**M**) and the actin filament skeleton in expansion on the substrate (**N**). (**O**) Filopodium formation is tagged with a purple colour. The open-source image processing software ImageJ [version ImageJ2 2.9.0/1.53t] was used for image analysis [47].

The reorganisation of the actin cytoskeleton is considered a fundamental mechanism in numerous cell metabolic activities, including those that involve structural modifications, proliferation, and differentiation. In particular, actin rearrangement plays a fundamental role in both cell adhesion and cellular spreading [46]. Therefore, the actin distribution on the cells that were grown on the substrates was studied using a controlled low-strength material (CLSM) and a combination of 2D and 3D images. As shown in Figure 8, the actin filaments appeared to be regularly organised as stress fibres running in parallel at the cell body level and in the cellular extension, which indicates that the substrates did not alter the actin assembly. In addition, multiple adhesion points (white arrows) and protrusions were revealed, which demonstrates the efficacy of the substrates in favouring the establishment of cell–cell and cell–substrate junctions (Figure 8).

## 4. Discussion

The concepts of both personalised medicine [33] and overcoming healthcare inequalities [6,48] have become extremely popular in recent decades. Polymers represent a perfect compromise as they enable cost reduction and the simplicity of a customised 3D printing process and they may lead to future wide-scale expansion. The need to reach underdeveloped markets is supported by research on plastic materials.

However, ensuring equal access to healthcare through the development of less expensive treatments and targeted solutions cannot be dissociated from minimising the impact on the planet. Indeed, achieving an ethical and ecological global healthcare system is an important challenge for the future [49].

From this perspective, samples of PCL+β-TCP 20% polymer were prepared in this study using a commercialised and well-established 3D printing system.

TCP is known to support bone augmentation, and the higher the percentage of polymers, the better osteoconductive support of the material for a ameliorate biological response [24]. However, a limit of conventional, commercialized, and low-cost 3D machines is the percentage of the TCP that can be incorporated into the polymeric phase. In this study, we identified the correct match between the two mentioned phases and to print devices with no alteration of their structure and geometry.

Indeed, the material flowed successfully through the core of the printer and could thus potentially be used by clinicians worldwide seeking to print their own medical devices. Due to the implant destination of the scaffold, its sterilisation is an essential aspect of safe surgical practices [50]. Single-use critical devices that come into contact with human tissues and blood must be decontaminated and sterilised before their employment, which is in accordance with European law 93/4236 [40] on medical meshes and scaffolds. Therefore, a material for biomedical applications that exhibits biocompatibility, bioresorbability, and biomimetic properties must remain stable during its sterilisation to be used for medical purposes [51,52].

However, although the sterilization of 3D printed critical devices is key for their safe usage, Tipnis et al. and Gulden et al. [50,53] recently stated that no standard methods for the sterilization of polymeric 3D printed materials had been developed. Essentially, the sterilization process must be optimised according to the material and the type of the device used.

Through a revision of the literature on the sterilisation of bedside 3D printed devices for use in operating rooms, Wiseman et al. [54] showed that peracetic acid and other low-temperature sterilization techniques that were analysed, such as 100% ethylene oxide, hydrogen peroxide, ozone, and gamma irradiation, exhibited ideal features for their use in routine preclinical sterilization approaches.

Immersion in peracetic acid is the most commonly used technique in the United States, and automated machines that use this compound are already commercially available. However, as suggested by Wiseman et al. [54], peracetic acid may be corrosive and has the potential to induce changes in the structural and biochemical properties of materials.

Our printable polymer showed no interactions between its ester double bonds and the peracetic acid employed in the sterilization. Conversely, although changes in the FT-IR spectrum of the materials were observed, notably, in the bands related to the -OH functional groups (3000–3600, 1500–1670, and below 700 cm^−1^), these were only related to water absorbance. Therefore, they were unlikely to represent any potentially harmful biological effects. In addition, no differences were found in the IR-ATR spectra of the PCL+β-TCP 20% samples.

In terms of mechanical tests, flexural static tests were conducted following the ISO 178 ASTM D790 standards [36] to identify the maximum deformation before rupture when applying a progressive force. The tests executed on the pure polymers yielded results in agreement with previously published data in terms of their tensile modulus and tensile strength [33]. No tensile tests were performed. The parameters tensile strength and tensile modulus were substituted for flexural strength and flexural modulus.

To evaluate whether the PCL characteristics were modified in the composite PCL+β-TCP 20%, the pure PCL samples were compared with the PCL composites. A tensile modulus of 0.2–0.5 GPa and tensile strength of 4–42 MPa were obtained for the PCL samples. The tests revealed that the PCL+β-TCP 20% samples exhibited a 0.3–0.4 GPa tensile modulus and 13–15 MPa tensile strength. Therefore, it could be deduced that the addition of the osteoconductive material did not change the mechanical behaviour of the composite in a statistically significant way. Moreover, our evidence suggests that the PCL+β-TCP 20% material withstood the chemical sterilization treatment and maintained consistent chemical–physical properties.

Additionally, in the interpretation of the mechanical properties, a relevant aspect to consider is that regardless of the different values of tensile modulus and other mechanical aspects between polymers and/or composite polymers and titanium, the devices used as scaffolds for periodontal bone regeneration do not have any support function [55].

This is the greatest difference between dental and maxillofacial surgeries and orthopaedic ones, where plaques, screws, and endoprosthesis must withstand different solicitations, such as compression and torsion [14,30]. Therefore, although the latest research efforts in the biomedical orthopaedic field have aimed to tailor materials with physical characteristics that are more similar to that of bone, the main goal in dental bone augmentation should be to contain the graft and protect bone growth throughout the mineralisation process with no stresses applied [12,56].

The main characteristic of a suitable material for tissue regeneration would be the absence of cytotoxic effects, combined with a capacity to allow cells to grow and colonise the substrate, as well as the deposition of an ECM over and inside the 3D scaffold meshes [57].

Our results show that the PCL+β-TCP 20% substrate supported cell growth within the 3D substrate; in particular, this biomaterial consented to the expansion of the cells into the “meshes” of the scaffold, which formed a 3D preosteoblast network characterised by a physical interconnectedness of the osteo-lineage cells via filopodia-like protrusions. The formation of these protrusions towards neighbouring cells is particularly important since they provide the cells with a possibility of sensing their surrounding environment and releasing osteo-building factors, thus reflecting the in vivo homeostatic behaviour of bone. It is well known that actin networks are characterised by tensed elastic strings and straws that are interconnected and control the fate of cells. Substrates with dissimilar mechanical characteristics affect the rearrangement of F-actin cytoskeleton in the cell maturation process and modulate peculiar cellular features, such as spreading, elasticity, and intramural signalling [58,59]. In this context, we observed that the stiffness of the PCL+β-TCP 20% substrate enhanced the stress fibre density and preosteoblast spreading.

Moreover, the MTS assay demonstrated that these substrates were biocompatible and showed no cytotoxicity. Indeed, they displayed an ability to induce cell viability and proliferation when compared with the culture plates.

The obtained results align with a recently published study by Akerlund et al. [34] in which a combination of PLA, PCL, and HA was investigated to develop customized biocompatible and bioresorbable polymer-based composite filaments, which finalised to faster healing. The authors also highlighted that the studied mineral accelerated polymer degradation, thus suggesting that the supports would only be required for a short period of time.

Therefore, according to the proliferation observed in our cultures, it is reasonable to hypothesise that in the early phase, the presence of an osteoconductive material could lead the cellular growth and further maturation, which, afterwards, would also fasten the resorption of the scaffold itself.

Another recent review by Jodati et al. [12] described the additional value of nanocomposites. These are crucial in the analysis of viable treatment strategies for bone tissue regeneration for specific bone defects such as craniofacial defects. In the review, the authors pointed out that composite ceramic compounds with added osteoconductive materials induced a greater adhesion of the bone cells on the scaffold.

Although there were no specific references about the timing of cell proliferation in the analysed literature, the results of our investigation suggest a very quick process of adhesion and proliferation. One possible reason for this positive outcome could also be related to the original roughness of the 3D-printed product.

Therefore, the PCL+β-TCP 20% scaffold showed the reliable role of osteoconductivity and provided a sort of network for the bone cells which could adhere and differentiate around the granules of the scaffold material due to the action of bone morphogenetic proteins.

## 5. Conclusions

In summary, the data presented here showed that the studied PCL+β-TCP 20% composite is satisfactory for commercial 3D printing and appears to be a suitable material to sustain an ISO14937:200935 sterilisation procedure.

In addition, the proper actin cytoskeleton rearrangement showed the biocompatibility of the material and its ability to favour osteoblast adhesion, which is a pivotal condition for cell proliferation and differentiation. The routine use of a reliable and low-cost polymer composite such as PCL+β-TCP 20% in private medical practice could help tackle global inequalities in oral health. Moreover, this material can be easily printed with an affordable 3D printer and can help avoid the need for a second surgery. However, these encouraging results should be further investigated through in vivo tests on animal models before being applied to humans.

## Figures and Tables

**Table 1:** Types of polymer and printing parameters.

Polymer	Printer	Nozzle Temperature	Print Bed	Printing Speed
**PCL**	Prusa Mini	110 °C	30 °C	60 mm/s
**PCL+β-TCP 20%**	Prusa Mini	110 °C	30 °C	60 mm/s

**Table 2:** Weight evaluation before and after the samples’ immersion in a solution of peracetic acid 2% for 12 min. The means ± the standard deviations were calculated for five samples from each group. No statistically significant differences were observed (*p* > 0.05).

Material	Weight Before (g)	Weight After (g)
**PCL**	2.11 ± 0.01	2.12 ± 0.01
**PCL+ β**-**TCP 20%**	2.26 ± 0.01	2.27 ± 0.01

**Table 3:** Three-point bending Zwick Roell tests on the studied poly(ε-caprolactone) (PCL) samples. All samples were evaluated before and after the sterilization processes. The means ± the standard deviations were calculated for five samples from each group. No statistically significant differences were observed (*p* > 0.05). Similarly, the tests were conducted on the studied poly(ε-caprolactone) (PCL) compounded with beta-tricalcium phosphate 20% (PCL+β-TCP 20%) samples. The means ± the standard deviations were calculated for five samples from each group. No statistically significant differences were observed (*p* > 0.05).

**PCL before Sterilization**				
	E_f_		s_fM_	e_fM_
	MPa		MPa	%
**PCL** (mean ± standard deviation)	338.5 ± 30.0		20.6 ± 1.0	11.4 ± 0.3
**PCL after sterilization**				
	E_f_		s_fM_	e_fM_
	MPa		MPa	%
**PCL** (mean ± standard deviation)	301.2 ± 19.6		19.4 ± 0.4	11.3 ± 0.2
**PCL+β-TCP 20% before sterilization**				
	E_f_		s_fM_	e_fM_
	Mpa		Mpa	%
**PCL+β-TCP 20%** (mean ± standard deviation)	382.1 ± 21.0		15.3 ± 0.5	9.5 ± 0.4
**PCL+β-TCP 20% after sterilization**				
	E_f_		s_fM_	e_fM_
	Mpa		Mpa	%
**PCL+β-TCP 20%** (mean ± standard deviation)	335.5 ± 6.9		14.5 ± 0.1	9.6 ± 0.1

## Data Availability

The data are available upon request from the authors.
